# dTtc1, a conserved tetratricopeptide repeat protein, is required for maturation of *Drosophila* egg chambers via its role in stabilizing electron transport chain components

**DOI:** 10.3389/fcell.2023.1148773

**Published:** 2023-06-02

**Authors:** Hannah Neiswender, Frederick C. Baker, Rajalakshmi Veeranan-Karmegam, Phylicia Allen, Graydon B. Gonsalvez

**Affiliations:** Cellular Biology and Anatomy, Medical College of Georgia, Augusta University, Augusta, GA, United States

**Keywords:** TRP domain, electron transport chain, Dynein, oocyte, mitochondria

## Abstract

We recently identified the *Drosophila* ortholog of TTC1 (dTtc1) as an interacting partner of Egalitarian, an RNA adaptor of the Dynein motor. In order to better understand the function of this relatively uncharacterized protein, we depleted dTtc1 in the *Drosophila* female germline. Depletion of dTtc1 resulted in defective oogenesis and no mature eggs were produced. A closer examination revealed that mRNA cargoes normally transported by Dynein were relatively unaffected. However, mitochondria in dTtc1 depleted egg chambers displayed an extremely swollen phenotype. Ultrastructural analysis revealed a lack of cristae. These phenotypes were not observed upon disruption of Dynein. Thus, this function of dTtc1 is likely to be Dynein independent. Consistent with a role for dTtc1 in mitochondrial biology, a published proteomics screen revealed that dTtc1 interacts with numerous components of electron transport chain (ETC) complexes. Our results indicate that the expression level of several of these ETC components was significantly reduced upon depletion of dTtc1. Importantly, this phenotype was completely rescued upon expression of wild-type GFP-dTtc1 in the depleted background. Lastly, we demonstrate that the mitochondrial phenotype caused by a lack of dTtc1 is not restricted to the germline but is also observed in somatic tissues. Our model suggests that dTtc1, likely in combination with cytoplasmic chaperones, is required for stabilizing ETC components.

## Introduction

The microtubule minus-end motor cytoplasmic Dynein (hereafter Dynein) is involved in transporting numerous types of cargo such as RNAs, proteins, vesicles, and organelles (Cason and Holzbaur, 2022; Jongsma et al., 2023). These cargos are typically linked to Dynein via adaptor proteins (Reck-Peterson et al., 2018; Canty and Yildiz, 2020). One such adaptor expressed in the *Drosophila melanogaster* oocyte and embryo is the RNA binding protein, Egalitarian (Egl) (Mach and Lehmann, 1997). Egl has been shown to link a growing list of RNAs with the Dynein motor (Dienstbier et al., 2009; Goldman and Gonsalvez, 2017; Vazquez-Pianzola et al., 2017; Goldman et al., 2019; Goldman et al., 2021). Localization of several of these mRNAs is key to establishment of polarity in the oocyte and to specification of the body plan in the embryo (Weil, 2014; Goldman and Gonsalvez, 2017).

In an effort to determine whether Egl also links non-RNA cargo with Dynein, we recently undertook a proteomics screen using *Drosophila* ovaries (Baker et al., 2021). One of the candidates identified in this screen was the uncharacterized gene CG14894. Because this gene is the fly ortholog of human TTC1/TPR1, we named the gene *dTtc1*. Very little is known regarding the function of dTtc1. Although the mammalian protein is similarly under-studied, it has been shown to be present in a complex with the heat shock protein, Hsp90 (Lotz et al., 2008). TTC1 contains tetratricopeptide repeats, a motif that is bound by C-terminal residues within Hsp90 (Scheufler et al., 2000). In addition, TTC1 also interacts with the A and B isoforms of VAP-33 (also referred to as VAPA and VAPB) (Lotz et al., 2008). Studies suggest that a trimeric complex consisting of TTC1, VAP-33, and Hsp90 is involved in vesicle trafficking (Lotz et al., 2008). This complex appears to be conserved in flies as dTtc1 was also found to interact with the fly orthologs of Hsp90 and VAP-33 (Baker et al., 2021).

Our interest in dTtc1 was spurred by the finding that TTC1 was identified in mammalian cells as a component of the Dynein complex (Redwine et al., 2017). We therefore hypothesized that dTtc1 might serve as a cargo or cargo adaptor of the Dynein motor. Surprisingly, however, we found that Dynein-localized mRNAs were not affected upon depletion of dTtc1. However, mitochondria in dTtc1 depleted egg chambers were significantly compromised; their numbers were reduced, they displayed a swollen morphology, and were largely devoid of cristae. Furthermore, the expression of numerous components of electron transport chain (ETC) complexes was greatly reduced in dTtc1 depleted ovaries. This phenotype is not germline specific, because depletion of dTtc1 in somatic tissues is also associated with similar mitochondrial defects. Thus, although our results do not rule out a potential function for dTtc1 in Dynein-related processes, our findings suggest a novel and primary role for dTtc1 in maintaining healthy mitochondria.

## Results

### dTtc1 is required for formation of the egg chamber

**FIGURE 1:**
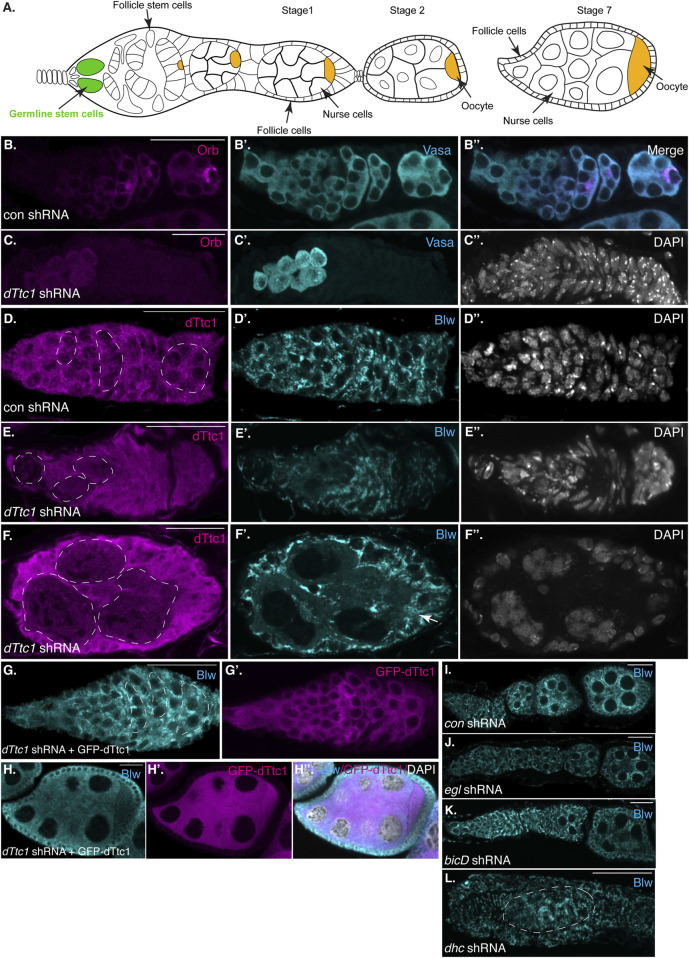
Early-stage depletion of dTtc1. **(A)** Schematic of a *Drosophila* female germarium as well as stage 1, 2 and 7 egg chambers. The germline stem cells are colored in green and the oocyte is shown in orange. **(B, C)** Ovaries were dissected and fixed from strains expressing either a control shRNA **(B)** or an shRNA against *dTtc1* **(C)**. The shRNAs were expressed using a driver that is active in the germarium. The egg chambers were processed for immunofluorescence using an antibody against Orb (magenta) and Vasa [cyan, **(B′, B′′, C′)**]. The egg chambers were also counterstained using DAPI to visualize nuclei [greyscale, **(C′′)**]. Depletion of dTtc1 results in defective formation of a 16-cell cyst. **(D–F)** The same control **(D)** and dTtc1 depleted egg chambers **(E, F)** were processed for immunofluorescence using antibodies against dTtc1 (magenta) and Blw [cyan, **(D′, E′, F′)**], a mitochondrial marker protein. The egg chambers were counterstained with DAPI to visualize nuclei **(D′′, E′′, F′′)**. Germline cysts are marked with a dashed line. dTtc1 depleted germline cells were almost completely devoid of mitochondria. By contrast, the surrounding somatic follicle cells contained normal mitochondria (arrows). **(G, H)** Ovaries were dissected and fixed from a strain co-expressing the *dTtc1* shRNA and a GFP-dTtc1 transgene. The egg chambers were processed for immunofluorescence using an antibody against Blw (cyan). The channel for GFP-dTtc1 is shown in **(G′, H′)** (magenta). **(H′′)** is a merged image of Blw, GFP-dTtc1 and the DAPI channel (DAPI shown in greyscale). Expression of GFP-dTtc1 rescued the mitochondrial phenotypes. **(I–L)** Ovaries were dissected and fixed from strains expressing shRNAs against eb1(I, control shRNA) *egl* **(J)**, bicD **(L)** or dhc **(L)**. The ovaries were processed for immunofluorescence using an antibody against Blw. Depletion of these components does not produce the same mitochondrial defect as observed upon dTtc1 depletion. The scale bar in these images corresponds to 20 microns.

The *Drosophila* egg chamber is composed of fifteen nurse cells and a single oocyte. Formation of the egg chamber involves division of a germline stem cell to produce a daughter cell known as a cystoblast. The cystoblast divides four more times to produce a sixteen-cell cyst. One of these cells will be specified as the oocyte, while the remainder differentiate as supportive cells known as nurse cells (Figure 1A) (Huynh and St Johnston, 2004).

Oocyte specification is a complex process involving numerous factors, including several components and regulators of the microtubule minus-end motor, Dynein (Suter and Steward, 1991; Carpenter, 1994; McGrail and Hays, 1997) (Figure 1A). Among these factors are Bicaudal-D (BicD), a Dynein activating adaptor, and Egalitarian (Egl), an RNA binding protein (Dienstbier et al., 2009; Hoogenraad and Akhmanova, 2016). The current model suggests that Egl binds numerous mRNAs in the *Drosophila* oocyte and embryo and links these mRNAs with Dynein via its interaction with BicD (McClintock et al., 2018; Sladewski et al., 2018; Goldman et al., 2019). However, it is also possible that Egl links non-RNA cargo with the Dynein motor. In order to explore this potential function of Egl, we undertook a proteomics screen using *Drosophila* ovaries to identify interacting partners of Egl. One candidate that was identified in this screen was the *Drosophila* ortholog of human TTC1, which we refer to as dTtc1 (Baker et al., 2021). We demonstrated in a recent publication that dTtc1 was enriched within the oocyte of stage 4 to stage 7 *Drosophila* egg chambers in an Egl-dependent manner (Baker et al., 2021) (Supplementary Figure S1A, arrows).

Relatively little is known regarding the function of dTtc1. However, human TTC1 was also identified in an independent proteomics screen as an interacting partner of Dynein light intermediate chain, as well as an interacting partner of BicD2, the human homolog of *Drosophila* BicD (Redwine et al., 2017). Thus, the association of TTC1 with the Dynein motor complex appears to be conserved between flies and mammals.

In order to identify the molecular function of dTtc1 we depleted this protein in the *Drosophila* germline. Immunofluorescence analysis indicates that the shRNA is capable of depleting endogenous dTtc1 and that the depletion is specific to germline cells (Supplementary Figures S1A, B). We initially used a driver that is active in the germanium, at the earliest stages of egg chamber formation. For simplicity, we will refer to this driver as a “germarium driver” (see Materials and methods for details). In contrast to strains expressing a control shRNA, strains expressing the shRNA against *dTtc1* contained extremely small degenerating ovaries (Supplementary Figure S1C, D). We examined dissected ovaries using an antibody against Orb, a marker for oocytes specification. Control egg chambers contained a single cell that was highly enriched for Orb (Figure 1B). By contrast, strains expressing the *dTtc1* shRNA displayed minimal Orb staining (Figure 1C). A closer examination using an antibody against Vasa, a protein expressed in germ cells, revealed that dTtc1 depleted strains failed to even form a sixteen-cell cyst (Figures 1B′, B″, C′, C″). Egg chamber formation involves a specialized membranous branched structure that connects germline stem cells and their differentiating progeny referred to as the fusome (Lin et al., 1994). As expected, the fusome was readily detected in egg chambers expressing a control shRNA (Supplementary Figure S2A, arrows). By contrast, the fusome was not formed in dTtc1 depleted egg chambers (Supplementary Figures S2B, B′). Thus, dTtc1 is required at the earliest stages within the germanium for formation of the egg chamber.

In a previous interactome analysis, dTtc1 was shown to interact with numerous mitochondrial proteins (Baker et al., 2021). We therefore examined control and dTtc1 depleted egg chambers using an antibody against Blw, a mitochondrial marker protein and the fly homolog of human ATP5A1. Rather astonishingly, cells within dTtc1 depleted cysts contained relatively few mitochondria (Figures 1D–F, dashed circles). By contrast, the surrounding somatic follicle cells, within which the shRNA is not expressed, displayed normal mitochondrial numbers and morphology (Figures 1E, F, arrow).

In order to test the specificity of this phenotype, we expressed GFP-dTtc1 in these egg chambers. Importantly, the dTtc1 used in this transgenic strain contained silent mutations that makes it refractory to the shRNA. Thus, using this strategy, we are able to deplete endogenous dTtc1 and express a wild-type version of GFP-dTtc1 in the same cells. Co-expressing wild-type GFP-dTtc1 and the *dttc1* shRNA in the germarium completely rescued these phenotypes. Egg chamber maturation occurs normally in these strains and mature oocytes were produced (Figures 1G, H). In addition, these egg chambers contained abundant mitochondria that were morphologically normal (Figures 1G, H). Based on these results, we conclude that the egg chamber degeneration and mitochondrial phenotypes are specifically caused due to depletion of dTtc1.

As noted previously, dTtc1 was identified as an interacting partner of Egl, a Dynein RNA adaptor. Loss of Egl and BicD in the germanium results in defective oocyte specification (Theurkauf et al., 1993). However, mitochondrial defects have not been reported in *egl* and *bicD* null mutants. Consistent with these previous studies, we found that depletion of Egl and BicD using the same germarium driver that was used to deplete dTtc1 produced morphologically normal egg chambers that were lacking an oocyte. Furthermore, we did not observe any overt mitochondrial phenotype in these depleted egg chambers (Figures 1I, J). Depletion of Dynein heavy chain (Dhc), the motor subunit of Dynein, resulted in a much more severe phenotype (Figure 1K). This is also consistent with prior studies indicating a role for Dynein in formation of the sixteen-cell cyst (McGrail and Hays, 1997). However, even in these aberrant egg chambers, the mitochondria appeared relatively normal (Figure 1K). Thus, in contrast to dTtc1 depletion, depletion of Dynein components at these earliest stages of oogenesis does not result in significant mitochondrial loss.

### Depletion of dTtc1 in mid-stage egg chambers also results in defective mitochondria

**FIGURE 2:**
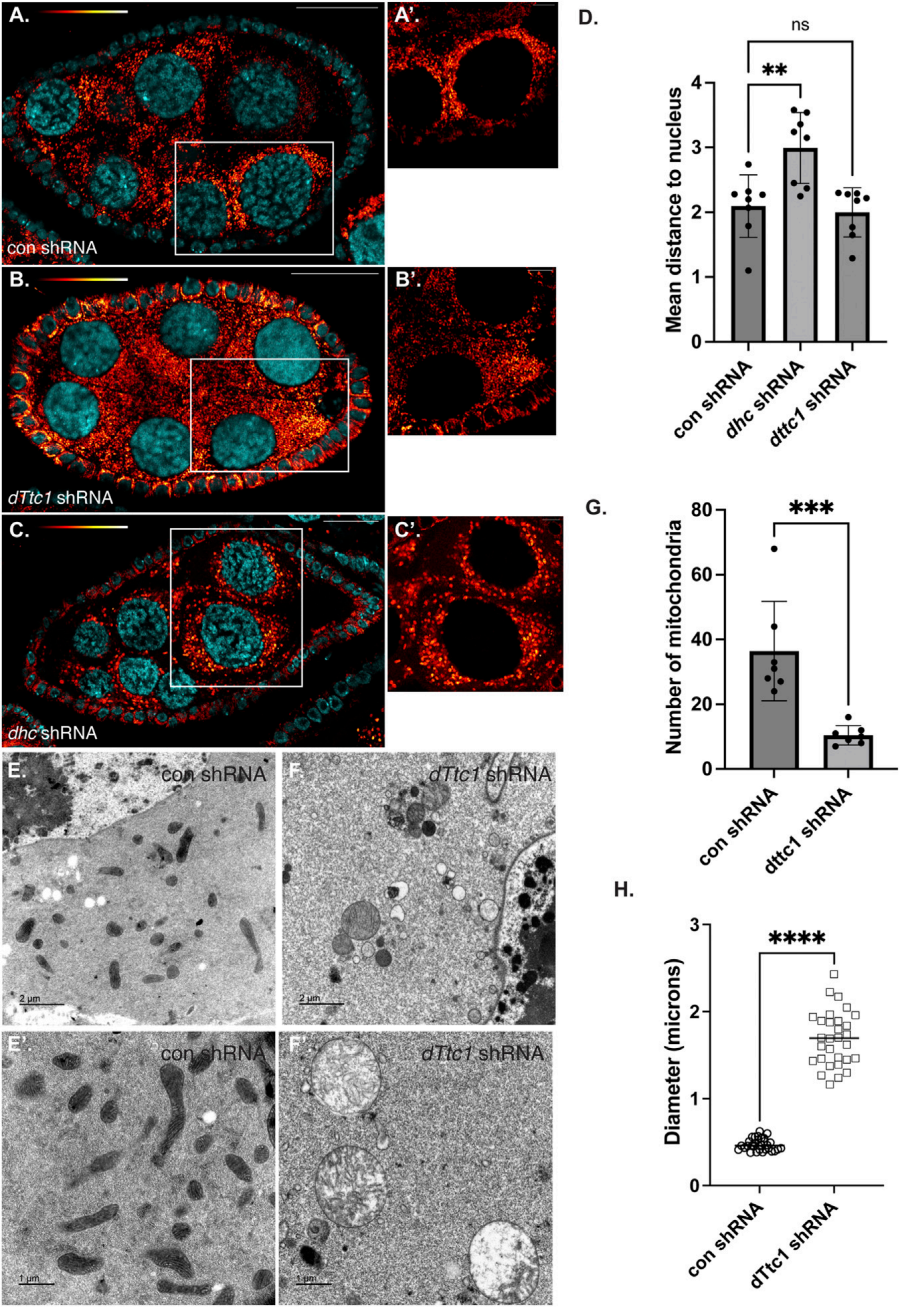
Mid-stage depletion of dTtc1. **(A–D)** Ovaries were dissected and fixed from strains expressing a control shRNA **(A)** an shRNA against *dhc* **(B)** or an shRNA against *dTtc1* **(B)** using a driver that is not active in the germanium but is active in early and mid-stage egg chambers (referred to in the Methods as an early-stage driver). The tissues were processed for immunofluorescence using an antibody against Blw (red to white LUT). **(A′–C′)** represent magnified images of the area shown in the white box. Mitochondria cluster in the vicinity of nurse cell nuclei. This clustering phenotype was disrupted in Dhc depleted egg chambers **(D)**. Mitochondrial clustering was not affected in dTtc1 depleted egg chambers **(D)**. However, these egg chambers contained fewer mitochondria that displayed a swollen phenotype. A one-way Anova was used for this analysis; **p ≤ 0.01, ns = not significant, *n* = 8 egg chambers. **(E–H)** The same control **(E)** and dTtc1 depleted ovaries **(F)** were processed for transmission electron microscopy. In comparison to the control, dTtc1 depleted egg chambers contained fewer mitochondria **(G)**. Note that **(E′,F′)** are from different areas of the slide and do not represent magnified images of the panels in E and **(F)**. An unpaired *t*-test was used for statistical analysis; ***p ≤ 0.001, *n* = 7 individual frames. **(H)** The mitochondrial diameter was determined for control and dTtc1 depleted strains using EM images. Consistent with what was observed using light microscopy, mitochondria in dTtc1 depleted egg chambers are enlarged. An unpaired *t*-test was used for statistical analysis; ****p ≤ 0.0001. *n* = 30 mitochondria were analyzed for each genotype. The scale bar in **(A–C)** is 20 microns; in **(A′–C′)** the scale bar is 5 microns; in **(C,D)** the scale bar is 2 microns; and in **(C′,D′)** the scale bar is 1 micron.

Loss of dTtc1 in the germanium results in extremely small ovaries that are difficult to fully characterize. We therefore expressed the *dttc1* shRNA using a driver that is active at slightly later stages of oogenesis (Sanghavi et al., 2016). For simplicity, we refer to this driver as an early-stage driver (see Materials and methods for details). Using this approach, dTtc1 function in the germanium is preserved, and egg chambers containing a single oocyte can be specified. However, even using this approach, mature eggs are not produced (Supplementary Figure S1, E, F). Consistent with published results (Lu et al., 2022), a similar phenotype was observed upon depleting Dhc using this early-stage driver (Supplementary Figure S1G). We therefore examined dTtc1 and Dhc depleted mid-stage egg chambers using a mitochondria marker antibody. In egg chambers expressing a control shRNA, mitochondria were abundant and were typically clustered in the vicinity of the nurse cell nuclei (Figures 2A, A′, D). However, this clustering phenotype was disrupted in Dhc depleted egg chambers (Figures 2B, D). Despite a lack of clustering, overall mitochondria numbers and morphology appeared unaffected (Figure 2B′; Supplementary Figure S2F). By contrast, egg chambers expressing the *dTtc1* shRNA contained fewer mitochondria (Figure 2C; Supplementary Figure S2F). In addition, the mitochondria that were present displayed a swollen and enlarged phenotype (Figure 2C′). Similar results were obtained using a strain expressing mitochondria labeled with EYFP (Supplementary Figures S2C, D, D′). However, despite the reduced number of mitochondria and their swollen appearance, clustering of mitochondria adjacent to nurse cell nuclei was preserved (Figures 2C, D). We therefore conclude that dTtc1 is required for maintaining adequate mitochondria numbers and normal morphology but not for their localization within the egg chamber. Dynein on the other hand, appears to be mostly involved in mitochondrial positioning.

In order to better understand the nature of the mitochondrial defect, and to examine these phenotypes with greater resolution, we processed control and dTtc1 depleted egg chambers for transmission electron microscopy. As expected, control egg chambers contained numerous mitochondria that were elongated with abundant cristae (Figures 2E, E′). Quantification of EM images revealed that mitochondria in dTtc1 depleted egg chambers were much less abundant, were enlarged, and largely devoid of cristae (Figures 2F, F′; Supplementary Figure S2G). Thus, depletion of dTtc1 in mid-stage egg chambers results in morphologically abnormal mitochondria.

**FIGURE 3:**
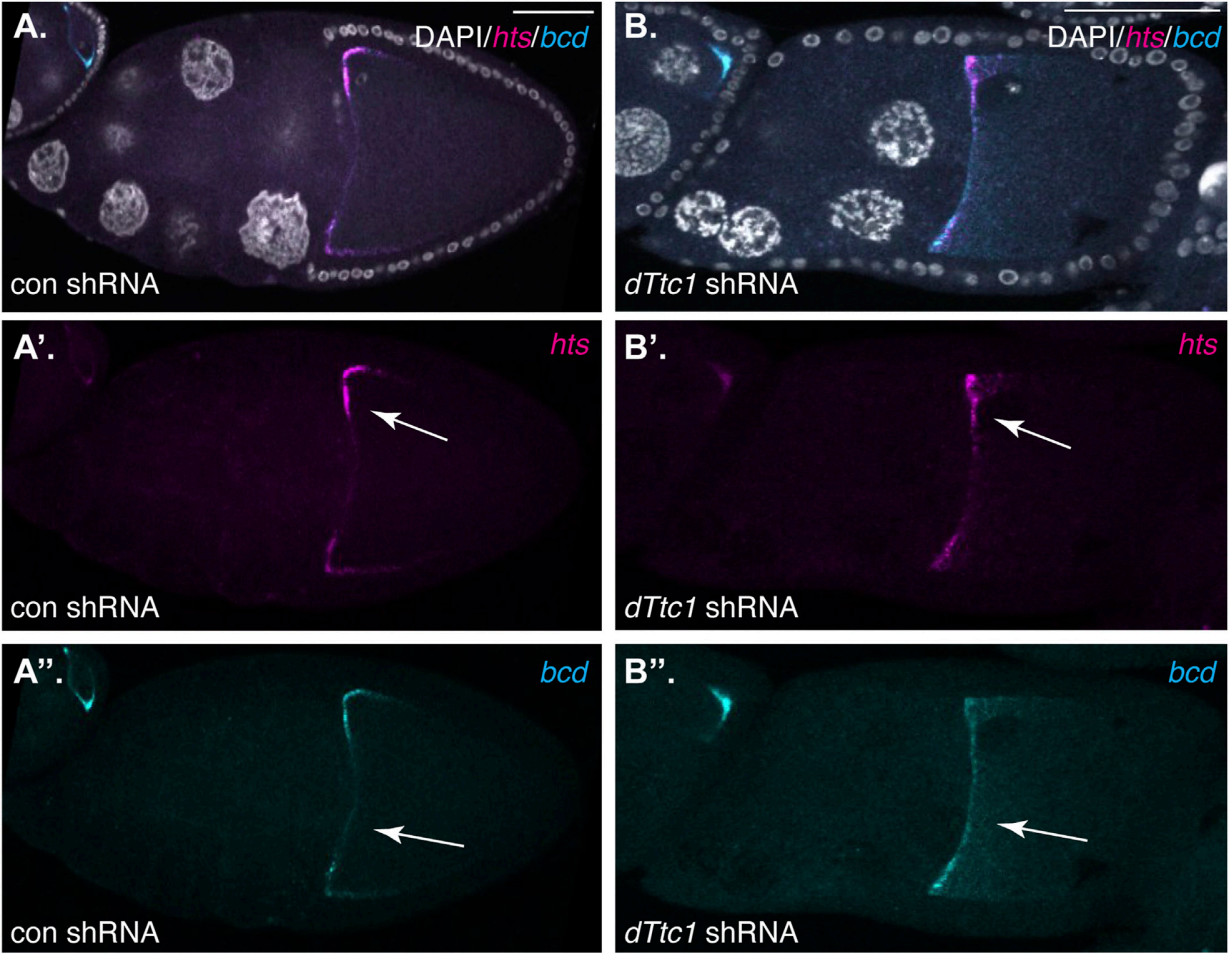
**(A, B)** Ovaries from flies expressing a control shRNA **(A)** or an shRNA against *dTtc1* **(B)** using the early-stage driver were dissected, fixed and processed for *in situ* hybridization using probes against *hts*(magenta) and *bcd* (cyan). The egg chambers were also counterstained with DAPI to visualize nuclei (greyscale). The individual channels for hts and bcd are shown in **(A′, B′, A′′, B′′)**. The anterior localization of these mRNAs was not affected by the depletion of dTtc1 (arrows).

The Dynein motor, Egl, and BicD are required for localization of several mRNAs such as *hts* and *bcd* in mid-stage egg chambers. In order to determine whether dTtc1 is required for these Dynein-mediated processes, we examined control and dTtc1 depleted egg chambers using *in situ* hybridization. In contrast to what is observed upon depletion of Egl (Goldman et al., 2021), *hts* and *bcd* remained correctly localized in dTtc1 depleted egg chambers (Figures 3A, B). Thus, although dTtc1 was identified as an interacting partner of Egl (Baker et al., 2021), depletion of dTtc1 does not appear to compromise Egl and Dynein-mediated mRNA localization.

### dTtc1 depletion results in reduced expression of electron transport chain components

**FIGURE 4:**
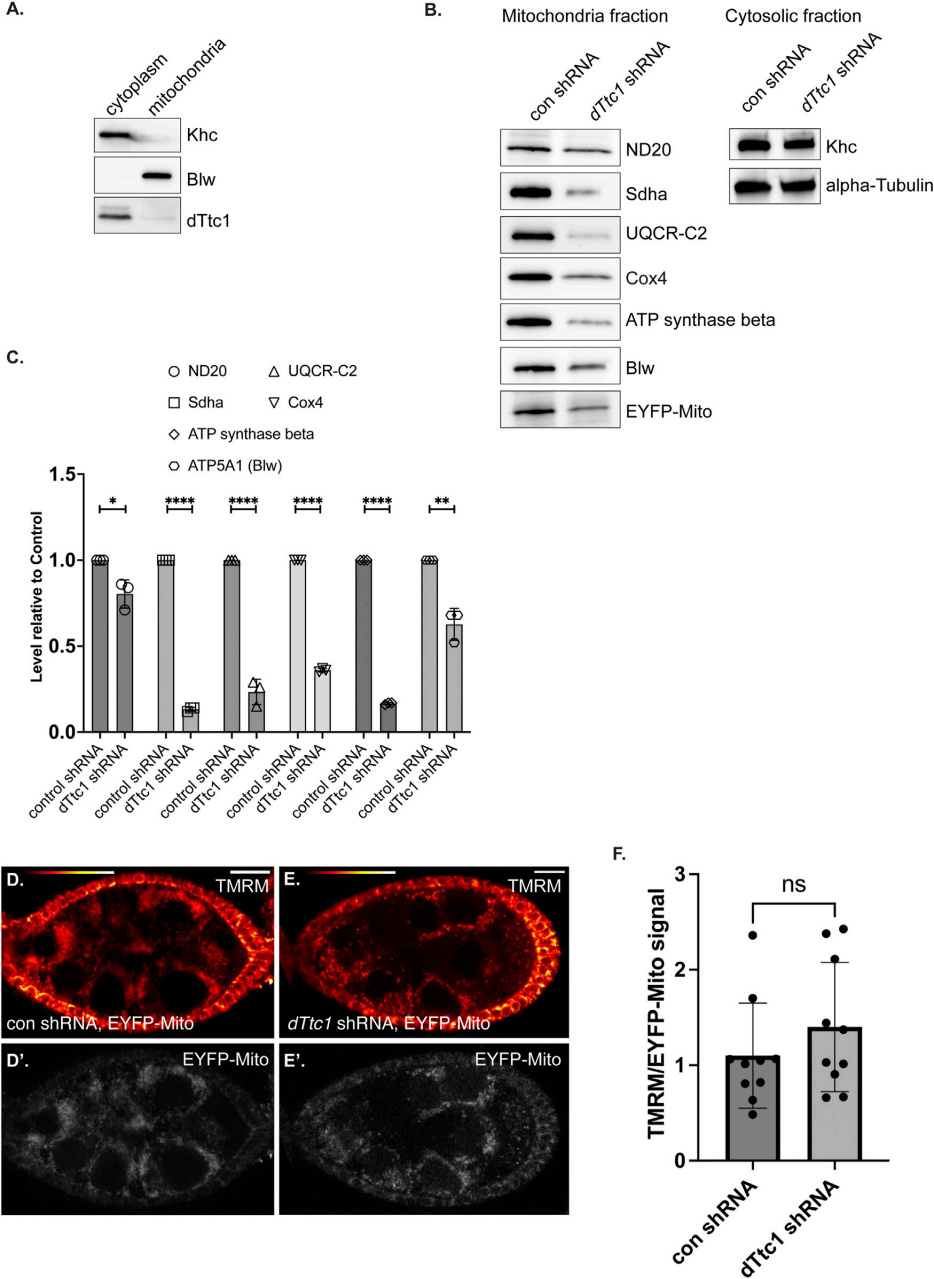
Electron transport chain components are expressed at a reduced level in dTtc1 depleted egg chambers. **(A)** Cytoplasmic and mitochondrial fractions were prepared from wild-type fly ovaries and were analyzed by western blotting using the indicated antibodies. dTtc1 is mostly present in the cytoplasmic fraction. **(B, C)** Cytoplasmic and mitochondrial fractions were prepared from ovaries of flies expressing either a control shRNA or an shRNA against *dTtc1* using the early-stage driver. The flies co-expressed mitochondria-targeted EYFP. The fractions were analyzed by western blotting using the indicated antibodies **(B)**. The results from three independent biological replicates were quantified and graphed **(C)**. An unpaired *t* test was used for statistical analysis; ****p ≤ 0.0001, **p ≤ 0.01, *p ≤ 0.05. **(D–F)** Live ovaries from the same strains were processed for TMRM labeling. After labeling, the samples were washed in PBS and imaged live on a Leica LSM780 inverted microscope. Signal for TMRM is shown using the red to white LUT **(D, E)** and signal for EYFP is shown using greyscale **(D′, E′)**. The graph indicates the ratio of TMRM signal to EYFP signal. An unpaired *t* test was used for statistical analysis; ns = not significant. *n* = 10 egg chambers for each genotype.

Given the mitochondrial phenotype observed upon depletion of dTtc1, we wondered whether dTtc1 localized to mitochondria. Egg chambers were processed for immunofluorescence using the dTtc1 antibody and a mitochondria marker antibody. High-resolution imaging did not reveal extensive colocalization between dTtc1 and mitochondria (Supplementary Figure S2E). Rather, dTtc1 appeared to be mostly cytoplasmic with occasional foci observed adjacent to mitochondria (Supplementary Figure S2E). Consistent with these results, fractionation of ovarian lysates revealed that dTtc1 was mostly present in the cytoplasmic fraction, with perhaps a small amount of the protein also present in the mitochondrial fraction (Figure 4A).

Previous studies have shown that dTtc1 interacts with several components of mitochondrial ETC complexes (Supplementary Figure S3A) (Baker et al., 2021). We therefore examined mitochondrial fractions by Western blotting using antibodies against Nd20, Sdha, UQCR-C2, Cox4, ATP synthase beta, and Blw (Figure 4B). Because of the small ovaries present in dTtc1 depleted females, we dissected approximately 1.5x more females from this strain in comparison to the control. In addition, the load was normalized to the total level of protein in the cytoplasmic fraction. As a further validation of equivalent loading, we also examined mitochondrial fractions by silver staining (Supplementary Figure S3B). Under these conditions, we observed that the level of all tested ETC components was reduced to varying degrees (Figures 4B, C). For instance, the level of ND20 was only modestly reduced, whereas the level of Sdha was dramatically reduced (Figures 4B, C). We did not observe an accumulation of these proteins in the cytosolic fraction (data not shown). Thus, the reduced level of these components does not appear to be due to defective mitochondrial import. Expression of GFP-dTtc1 in this background completely rescued this phenotype (Supplementary Figure S3C). Collectively, these results indicate that depletion of dTtc1 in the germline results in mitochondria with defective morphology and reduced expression of ETC components.

We next examined mitochondrial function in these tissues. Live egg chambers from strains expressing an EYFP-mito marker as well as either a control shRNA or an shRNA against *dTtc1* were processed using TMRM. This dye is incorporated into mitochondria dependent upon membrane potential (Garcez et al., 2020). In order to normalize the signal to total mitochondria, the ratio of TMRM signal was compared to the signal for EYFP-mito. Using this strategy, we did not observe a significant difference in mitochondrial membrane potential between the control and dTtc1 depleted strains (Figures 4D, E). Thus, despite the reduced mitochondrial numbers and morphology differences, the membrane potential in mitochondria in dTtc1 depleted egg chambers does not appear to be affected. However, there might be other aspects of mitochondrial function that are defective in dTtc1 depleted egg chambers. Further experimentation will be required to evaluate this.

### The mitochondrial role of dTtc1 is not restricted to the germline

**FIGURE 5:**
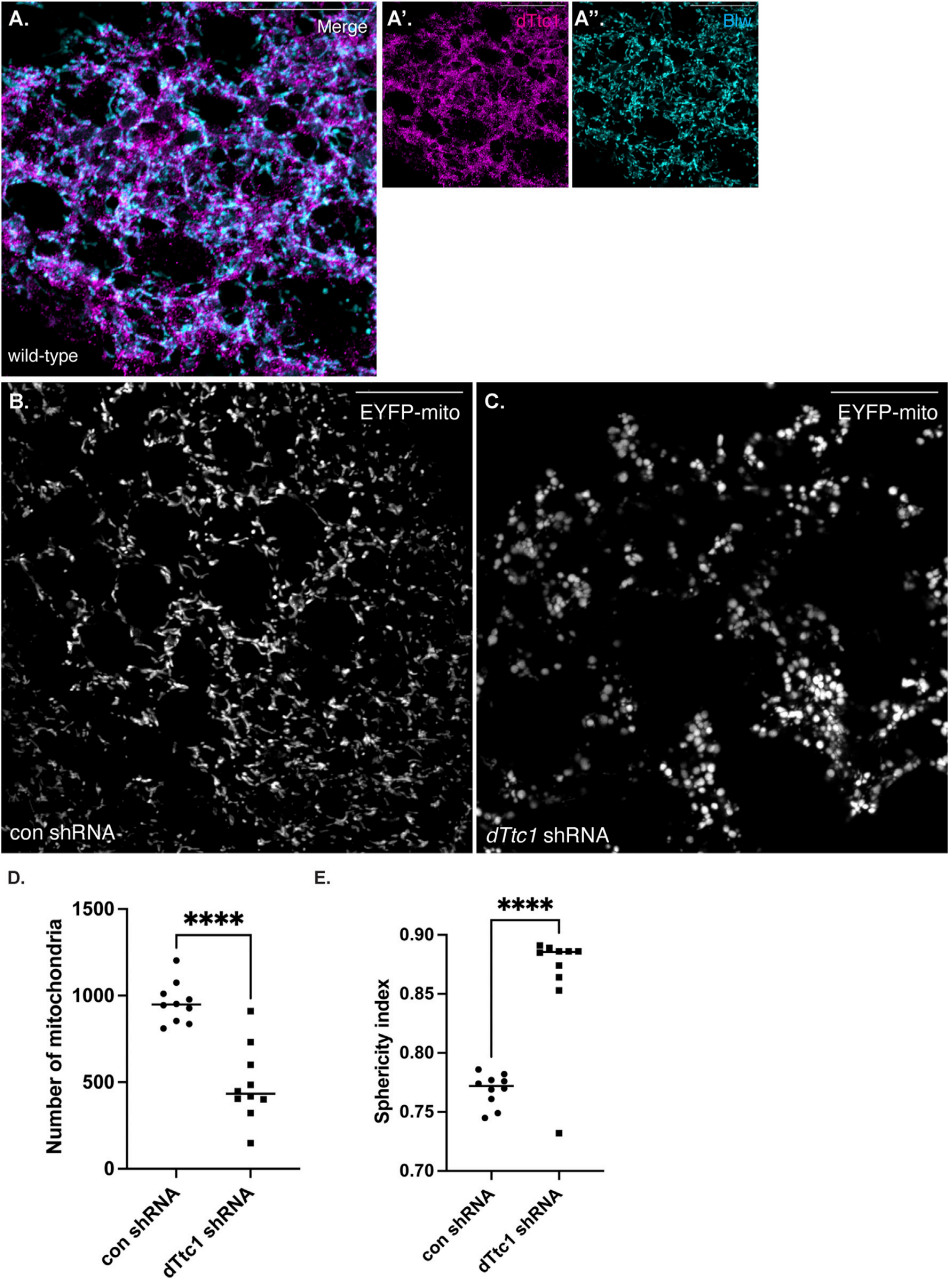
dTtc1 is also required for maintaining mitochondrial numbers and morphology in somatic tissues. **(A)** Larval fat body tissue was dissected and processed for immunofluorescence using antibodies against dTtc1 [magenta, **(A′)**] and Blw [cyan, **(A′′)**]. dTtc1 does not colocalize with mitochondria, but rather is present in foci that are often adjacent to mitochondria. **(B–E)** A strain co-expressing GFP targeted mitochondria and a driver that is active in fat body cells was crossed to a strain expressing a control shRNA **(B)** or an shRNA against *dTtc1* **(C)**. Fat body tissue was dissected from the resulting larvae, fixed and processed for microscopy. In comparison to flies expressing a control shRNA **(B)**, flies expressing the *dTtc1* shRNA **(C)** have reduced numbers of mitochondria **(D)**. In addition, the sphericity index of mitochondria in dTtc1 depleted fat body cells was also higher than the control. This indicates that the mitochondria in dTtc1 depleted cells were less elongated and were more spherical. The scale bar in these images corresponds to 20 microns. An unpaired *t* test was used for statistical analysis; ****p ≤ 0.0001. For **(D)**, 10 individual frames were analyzed for each genotype. The same images were used for the quantification in **(E)**. For this analysis, a total of 9591 mitochondria were scored in the control shRNA background and 4871 mitochondria were scored in the *dTtc1* shRNA background. The plotted sphericity index corresponds to the mean value for each of the 10 individual frames.

Although our initial studies were performed using female germline tissue, dTtc1 appears to be widely expressed (Gerstein et al., 2014; Leader et al., 2018). Larval fat bodies are often used as a model somatic tissue for the study of mitochondrial function (Zhou et al., 2019). We therefore examined the expression and localization of dTtc1 in these cells. As expected, specific signal for dTtc1 could be detected in fat bodies and co-localization analysis revealed that foci of dTtc1 could often be detected adjacent to mitochondria (Figure 5A). We next depleted dTtc1 in these cells using an shRNA and a driver that is active in fat bodies (Zhou et al., 2019). Consistent with what was observed in the egg chamber, depletion of dTtc1 in fat bodies resulted in significantly reduced mitochondrial content (Figures 5B–D). In addition, rather than the longer and filamentous mitochondria observed in control strains, mitochondria in dTtc1 depleted egg chambers displayed a swollen appearance (Figures 5B, C). Consistent with this observation, the sphericity index (see Materials and methods for details) of mitochondria in dTtc1 depleted fat bodies was higher than what was observed in the control strain (Figure 5E). The sphericity index is a measure of how spherical an object is. As such, mitochondria that display a round and swollen appearance will have a higher sphericity index that mitochondria that have a more filamentous morphology. Lastly, bright field imaging revealed that the overall morphology of the fat body was affected upon depletion of dTtc1 (Supplementary Figures S4A, B).

In summary, our results indicate that dTtc1 is a cytosolic protein that plays an important role in controlling the expression level of several ETC components. As a result of this function, depletion of dTtc1 results in morphologically abnormal mitochondria in germline and somatic tissues.

## Discussion

In this report, we describe the first characterization of dTtc1 function in germline and somatic tissues using the *Drosophila* egg chamber and fat body cells as models. dTtc1, like its human ortholog, TTC1/TPR1, is a tetratricopeptide repeat (TPR) containing protein. TRP repeats are often involved in mediating protein-protein interaction (Perez-Riba and Itzhaki, 2019). We identified dTtc1 as an interacting partner of Egalitarian (Egl), an RNA adapter for the Dynein motor. Interestingly, the interaction between TTC1 and Dynein appears to be conserved between flies and mammals (Redwine et al., 2017; Baker et al., 2021). Our initial hypothesis was that dTtc1, and perhaps human TTC1, might be involved in linking cargo with Dynein. However, Dynein related processes such as localization of *hu li tai shao* (*hts*) and *bicoid* (*bcd*) mRNAs were unaffected upon depletion of dTtc1. The main defect in dTtc1 depleted tissues appears to be a mitochondrial deficit. Mitochondria numbers are reduced, they display an abnormal enlarged morphology, and the expression level of numerous ETC components is significantly reduced in dTtc1 depleted tissues. Importantly, these defects are rescued upon expression of wild-type GFP-dTtc1. Thus, these phenotypes are specifically due to a lack of dTtc1. Interestingly, depletion of certain ETC components has also been shown to produce a similar enlarged mitochondrial phenotype in fat body cells (Zhou et al., 2019).

In contrast to dTtc1 depletion, loss of either Egl or Dynein components was not associated with similar mitochondrial defects. However, Dhc depletion was associated with a defect in mitochondrial clustering adjacent to nurse cell nuclei. This is consistent with a role for Dynein in transport of mitochondria. Both Kinesin-1 and Dynein have been shown to be required for transporting mitochondria in *Drosophila* axons (Pilling et al., 2006). Both motors are linked to mitochondria via the protein Milton (or its human homologs TRAK1 and TRAK2) (Cox and Spradling, 2006; Fenton et al., 2021; Canty et al., 2023). However, the role of Kinesin-1 and Dynein in mitochondrial transport within the egg chamber is relatively unknown. Our results suggest that in the absence of Dhc, mitochondrial clustering adjacent to nurse cell nuclei is defective. However, from these data we cannot conclude whether this phenotype results from a lack of Dynein-mediated transport towards the nuclei or whether this results from excessive Kinesin transport away from nuclei. Further studies will be required to differentiate between these scenarios.

Why does depletion of dTtc1 result in these profound mitochondrial phenotypes? At present, we do not have a definitive answer to this question. dTtc1 does not co-localize with mitochondria and fractionation suggests that most of the protein resides in the cytosol. Thus, dTtc1 does not appear to be a core mitochondrial protein. In addition, we do not see an obvious accumulation of ETC components in the cytosolic fraction upon depletion of dTtc1. Thus, dTtc1 is not likely to be directly involved in importing proteins into mitochondria. In addition, despite the abnormal morphology of mitochondria in dTtc1 depleted egg chambers, the membrane potential of the mitochondria appeared to be relatively unaffected.

A potential clue to the function of dTtc1 might reside in its conserved interaction with Hsp90. Hsp90 has been shown to bind to human TTC1 via its TRP domains and a proteomics screen identified the *Drosophila* ortholog of Hsp90 (Hsp83) as a potential interaction partner (Lotz et al., 2008; Baker et al., 2021). In addition to Hsp90, ten additional chaperones were also identified as potential interaction partners of dTtc1 in the same screen (Supplementary Figure S4C) (Baker et al., 2021). Based on these observations, we hypothesize that dTtc1 functions primarily as a cytosolic co-chaperone for the folding of key mitochondrial proteins. When dTtc1 is lacking, these mitochondrial proteins are not correctly folded and are subsequently degraded. It would be interesting to determine whether inhibiting proteasomal degradation in dTtc1 depleted flies restores the expression level of ETC components.

Our initial interest in dTtc1 was due to its conserved interaction with the Dynein complex. As noted above, depletion of dTtc1 does not produce overt phenotypes resembling a defect in Dynein mediated processes. However, given the conserved interaction between dTtc1 and Dynein components, it is possible that dTtc1 and its human ortholog perform a similar co-chaperone function for components of the Dynein motor. Dynein is a very large multi-subunit complex and certain chaperones such as the Prefoldin complex have been shown to interact with newly synthesized Dynein intermediate chains (Ozdemir et al., 2016). In addition, a component of the Chaperonin complex has also been shown to interact with the Dynactin1, a subunit of the large Dynactin complex, a critical regulator of Dynein activity (Echbarthi et al., 2018). Thus, it is possible that dTtc1 associates with Dynein components to assist in their correct folding. However, due to the redundancy of additional chaperones that also function in the folding of Dynein components, loss of dTtc1 might not result in a significant defect in Dynein mediated processes. By contrast, the role of dTtc1in the folding mitochondrial protein might be more central to its function, thus resulting in dramatic mitochondrial phenotypes when dTtc1 is lacking.

## Materials and methods

### Fly stocks

Endogenous dTtc1 was depleted in the female germline using *dttc1* shRNA expressed using the Valium22 vector (Bloomington stock center; #42763, donor TRiP) and in fat body cells using *dttc1* shRNA expressed using the Valium20 vector (Bloomington stock center; #53005, donor TRiP). For depletion of factors at the earliest stage of oogenesis, within the germarium, expression of the shRNA was driven using P{GAL4::VP16-nanos.UTR}CG6325[MVD1] (Bloomington Stock Center, #4937; donor Ruth Lehmann). We refer to this driver in the results section as a germarium driver. For early and mid-stage depletion, expression of the shRNA was driven using P{w[+mC] = matalpha4-GAL-VP16}V37 (Bloomington Stock Center, #7063; donor Andrea Brand). We refer to this driver in the results sections as the early-stage driver. Depletion of dTtc1 in fat body cells was driven using w[1118]; P{w[+mC] = Cg-GAL4.A}2 (Bloomington Stock Center, #7011; donor Chuck Dearolf). In addition, the following shRNA strains were also used: *egl* shNRA (Bloomington stock center; #43550, donor TRiP), *bicD* shRNA (Bloomington stock center; #35405, donor TRiP) and *dhc* shRNA (Bloomington stock center; #36583, donor TRiP). A strain using mitochondria targeted EYFP (P{sqh-EYFP-Mito}3, Bloomington stock center; #7194, donor Dennis LaJeunesse) was used to validate the swollen mitochondria phenotype. The GFP-dTtc1 used in the rescue experiments was generated in a previous publication (Baker et al., 2021). OR flies were used as the wild-type control. Fly crosses for all experiments were maintained at 25°C. For examination of ovarian phenotypes, female flies were dissected at 3 days of age. Third instar larvae were dissected for examining fat body phenotypes.

### Mitochondria fractionation

Ovaries were homogenized in an extraction buffer (5 mM Hepes, pH 7.5, 210 mM mannitol, 70 mM sucrose, and 1 mM EGTA). Next, the lysate was subjected to two rounds of centrifugation, both at 700 g for 5 min at 4°C. The supernatant from each step was transferred into a new microfuge tube. The resulting supernatant was then subjected to a final round of centrifugation at 9,000 g for 10 min at 4°C. The supernatant from this step contains the cytosolic fraction and the pellet contains the mitochondrial fraction. The fractions were mixed with Laemmli buffer, run on a gel and analyzed by Western blotting. All Western blot images were acquired on a Bio Rad ChemiDoc MP.

### Antibodies

The following antibodies were used for western analysis: rabbit anti-UQCR-C2 (III) (from Dr. Owusu-Ansah, 1:2,000), rabbit anti-COX IV (from Dr. Owusu-Ansah, 1:2,000), rabbit anti-Sdha(II) (from Dr. Owusu-Ansah, 1:1,500), rabbit anti-ND-20 (I) (from Dr. Owusu-Ansah, 1:2,000) (Murari et al., 2020), mouse anti-ATP synthase beta(V) (Life technologies, 1:2,000), mouse anti-ATP5A1 (Life technologies, 1:30,000 for western, 1:3,000 for immunofluorescence), mouse anti-GFP (Chromotek, 1:5,000), mouse anti-a-Tub (Millipore-Sigma, 1:60,000), rabbit anti-Khc (generated inhouse, 1:5,000), rabbit anti-dTtc1 (1:5,000 for western, 1:100 for immunofluorescence), mouse anti-Orb (Developmental studies hybridoma bank, 1:30 for immunofluorescence), rat anti-Vasa (Developmental studies hybridoma bank, 1:50 for immunofluorescence), and mouse anti-Spectrin (Developmental studies hybridoma bank, 1:20 for immunofluorescence). The following secondary antibodies were used: goat anti-rabbit Alexa 594, 555 and 488 (Life Technologies, 1:400, 1:400 and 1:400 respectively); goat anti-mouse Alexa 594, 555 and 488 (Life Technologies, 1:400, 1:400 and 1:400 respectively); goat anti-rat Alexa 488 (Life Technologies, 1:400); goat anti-mouse HRP (Pierce, 1:5000); and goat anti-rabbit HRP (Pierce, 1:5000).

### Immunofluorescence *in situ* hybridization and imaging

Immunofluorescence and RNA *in situ* hybridization on dissected ovaries was performed as described previously (Goldman et al., 2021; Neiswender et al., 2021). Dissected ovaries were fixed in 4% formaldehyde (Pierce) for 20 min at room temperature. For immunofluorescence experiments, the primary antibody was incubated in blocking buffer (PBS +0.1% Triton X-100 + 2% BSA) overnight at 4°C. Next, the samples were washed 3 times in PBST (PBS +0.1% Triton X-100) and incubated overnight with the fluorescent secondary antibody in the same blocking buffer. This was followed by 4 washes with PBST. The tissue was then stained with DAPI, and mounted onto slides using Prolong Diamond (Life technologies).

The fixation step for RNA *in situ* hybridization was the same as described above. After fixation, ovaries were stored in 100% methanol at −20°C for 1 h. Following this, the samples were re-hydrated with three 10 min washes using a solution of PBST and 100% methanol (3:7, 1:1, 7:3) and rinsed 4 times with PBST. Next, the samples were washed for 10 min in Wash Buffer (4xSSC, 35% deionized formamide, 0.1% Tween-20). Fluorescent oligo probes (Stellaris probes) were purchased from Biosearch technologies. Probes diluted in Hybridization Buffer (10% dextran sulfate, 0.1 mg/mL salmon sperm ssDNA, 100 µL vanadyl ribonucleoside (NEB biolabs), 20ug/mL RNAse-free BSA, 4x SSC, 0.1% Tween-20, 35% deionized formamide) were added to the ovaries and incubated overnight at 37°C. The next day, the samples were washed 2 times with pre-warmed wash Buffer for 30 min. After 2 rinses with PBST and 2 rinses with PBS, the samples were counter-stained with DAPI and mounted onto slides using Prolong Diamond.

Immunofluorescence on fat body tissue was also performed using a similar protocol. In brief, fat body tissue was isolated from L3 larvae in cold PBS and fixed with 4% formaldehyde for 40 min without agitation. After fixation, the samples were washed once with PBS and then permeabilized using PBS containing 0.2% Triton X-100. The tissue was incubated with primary antibody in blocking buffer (PBS +0.1% Triton X-100 + 2% BSA) overnight at 4°C. The next day, the samples were washed three times with PBST (PBS +0.1% Triton X-100) and incubated overnight with the fluorescent secondary antibody diluted into the same blocking buffer. Finally, the samples were washed four times with PBST, DAPI stained, and mounted onto slides using Prolong Diamond (Life technologies).

### Transmission electron microscopy

Dissected ovaries were processed for transmission electron microscopy as described previously (Liu et al., 2015). Briefly, ovarian tissue was fixed using 4% paraformaldehyde and 2% glutaraldehyde in 0.1 M sodium cacodylate (NaCac) buffer (pH 7.4). Ovarioles were then embedded within agarose. Stage 9 and 10 egg chambers were idenfied and isolated out of the agarose. The samples were post fixed in 2% osmium tetroxide in NaCac, stained with 2% uranyl acetate, dehydrated with a graded ethanol series, and embedded in EponAraldite resin. Next, thin sections were cut using a diamond knife on a Leica EM UC6 ultramicrotome (Leica Microsystems, Bannockburn, IL), collected on copper grids, and stained with uranyl acetate and lead citrate. Egg chambers were observed in a JEM 1230 transmission electron microscope (JEOL USA, Peabody, MA) at 110 kV and imaged with an UltraScan 4000 CCD camera and First Light Digital Camera Controller (Gatan, Pleasanton, CA).

### Light microscopy

Light microscopy Images were captured on either an inverted Leica Stellaris confocal microscope or an inverted Zeiss LSM780 equipped with Airyscan. Images were processed for presentation using Fiji, Adobe Photoshop, and Adobe Illustrator. All imaging experiments were performed at the Augusta University Cell Imaging Core.

### TMRM live imaging

Ovaries were dissected in Schneider’s media (Gibco) containing 15% fetal bovine serum (Corning). Dissected ovaries were incubated in 100 nM of Image-iT™ TMRM Reagent (Invitrogen) for 25 min. After the incubation period, the ovaries were washed 3 times with dissection medium, adhered to a poly lysine coated 35 mm glass bottom dish (1.5 coverslip, MatTek), and immediately imaged on a Zeiss LSM780 inverted microscope.

### Silver stain

Mitochondrial fractions were run on a 4%–15% gradient gel (BioRad). Proteins were visualized using the Pierce Silver stain kit following the directions provided by the manufacturer.

### Quantifications

Western blot images from the Biorad Chemidoc MP were quantified using the Image lab software from Biorad. Three independent biological replicates were used for quantification. The expression level of ETC components in the dTtc1 shRNA strain was compared to the level of the same protein in the control shRNA strain. The mean distance of the mitochondria to the nucleus was determined using Imaris 10.0. Nuclei were defined using the “surfaces” module and mitochondria were defined using the “spots” module in Imaris. These same data were used to determine the number of mitochondria in each of the indicated genotypes. The number of mitochondria reported in Figure 2 was manually counted using the transmission EM images. The diameter of mitochondria in the transmission electron microscopy images was quantified using Fiji. In order to determine membrane potential, the ratio of the TMRM signal to the EYFP signal was determined between control and dTtc1 depleted live egg chambers. The pixel intensity measurements for this figure were performed using Fiji. The mitochondria number and sphericity index of mitochondria in larval fat bodies were quantified using Imaris 10.0. For Figure 5, mitochondria were defined using the “surface” module of Imaris. This module enabled us to more precisely map the filamentous mitochondria morphologies found in the control strain. In addition, this module enabled us to calculate the sphericity index of mitochondria. In essence, this is an indicator of how spherical an object is and is defined as the ratio of the surface area of a sphere, with the same volume as the given object, to the surface area of the object. The following formula is used by Imaris to calculate the sphericity index:

Vp = volume of object

Ap = surface area of object

Cristae frequency was quantified using our EM images as described by Shi et al. (2022). It is defined as the number of cristae in a given mitochondria divided by the length of the mitochondria. Graphs were assembled using Graphpad Prism9. Statistical analysis was also performed using Prism9.

## Data Availability

The original contributions presented in the study are included in the article/Supplementary Material, further inquiries can be directed to the corresponding author.

## Supplementary material

SUPPLEMENTARY FIGURE S1
**(A,B)** Ovaries from flies expressing a control shRNA **(A)** or an shRNA against *dTtc1* **(B)** using the early-stage driver were dissected, fixed and processed for immunofluorescence using an antibody against dTtc1 (red to white LUT). The egg chambers were also counterstained with DAPI to visualize nuclei (cyan). Expression of *dttc1* shRNA results in the germline specific depletion of dTtc1. **(C,D)** Ovaries from flies expressing a control shRNA **(C)** or an shRNA against *dTtc1* **(D)** using the germarium driver were dissected, fixed and stained with DAPI (cyan). Entire ovaries were mounted and imaged. The scale bar in C and D is 100 microns. **(E–G)** Ovaries from flies expressing a control shRNA **(E)**, and shRNA against *dTtc1* **(F)**, or an shRNA against *dhc* **(G)** using the early-stage driver were dissected, fixed and stained with DAPI (cyan). Entire ovaries were mounted and imaged. The scale bar in E-F is 500 microns.

SUPPLEMENTARY FIGURE S2
**(A,B)** Ovaries from flies expressing a control shRNA **(A)** or an shRNA against *dTtc1* **(B)** using the germarium driver were dissected, fixed and stained using antibodies against Spectrin (cyan) and dTtc1 (greyscale) **(B′)**. The ovaries were counterstained with DAPI (magenta). Spectrin is a marker of the fusome. The fusome fails to form in dTtc1 depleted egg chambers. **(C, D)** Egg chambers from flies co-expressing mitochondria-targeted EYFP and either a control shRNA **(C)** or shRNA against *dttc1* **(D)** driven using the early-stage driver were fixed and imaged. Signal for EYFP is shown using a red to white LUT. Depletion of dTtc1 results in fewer mitochondria per egg chamber. In addition, the mitochondria in the dTtc1 depleted strain display a swollen phenotype **(D′)**. **(E)** Ovaries from wild-type flies were fixed and processed for immunofluorescence using antibodies against dTtc1 (magenta) and Blw (cyan). dTtc1 does not extensively co-localize with mitochondria. The scale bar in D′ is 5 microns. In the rest of the images, the scale bar is 20 microns **(F)**. The number of mitochondria in strains expressing either a control shRNA, an shRNA against *dhc* or an shRNA against *dTtc1* was analyzed using the “spots” module in Imaris. The early-stage driver was used to express the shRNAs. An unpaired *t*-test was used for statistical analysis; **p* ≤ 0.05, ns = not significant, *n* = 15 mitochondria per genotype. **(G)** The cristae frequency was calculated from EM images of egg chambers expressing either the control shRNA or an shRNA against *dTtc1*. The cristae frequency is defined as the number of cristae in each mitochondria divided by the length of the mitochondria (Shi et al., 2022). An unpaired *t*-test was used for statistical analysis; *****p* ≤ 0.0001, *n* = 15 mitochondria per genotype.

SUPPLEMENTARY FIGURE S3
**(A)** A list of the electron transport chain components that were shown to be part of the dTtc1 interactome (Baker et al., 2021). **(B)** Mitochondrial fractions from strains expressing a control shRNA or an shRNA against *dTtc1* were run on a gel and silver stained. These same fractions were also processed for the western blots shown in Figure 4B **(C)** Cytoplasmic and mitochondrial fractions were prepared from ovaries of flies expressing a control shRNA, an shRNA against *dttc1,* or co-expressing *dttc1* shRNA and GFP-dTtc1. Co-expression of GFP-dTtc1 rescues the expression level defect of Cox4 observed upon depletion of endogenous dTtc1.

SUPPLEMENTARY FIGURE S4
**(A,B)** Larval fat bodies were dissected from strains expressing a control shRNA **(A)** or from larvae expressing an shRNA against *dTtc1* **(B)**. Bright field images are shown. Depletion of dTtc1 results in abnormal morphology of the fat body (arrows). **(C)** A list of chaperone proteins that were shown to be part of the dTtc1 interactome (Baker et al., 2021). The scale bar in these images is 20 microns.
