# Supplementary figures and images for: dTtc1, a conserved tetratricopeptide repeat protein, is required for maturation of Drosophila egg chambers via its role in stabilizing electron transport chain components
Source: Front Cell Dev Biol. 2023 Jun 2;11:1148773. doi: 10.3389/fcell.2023.1148773 (PMC10272552; doi:10.3389/fcell.2023.1148773)

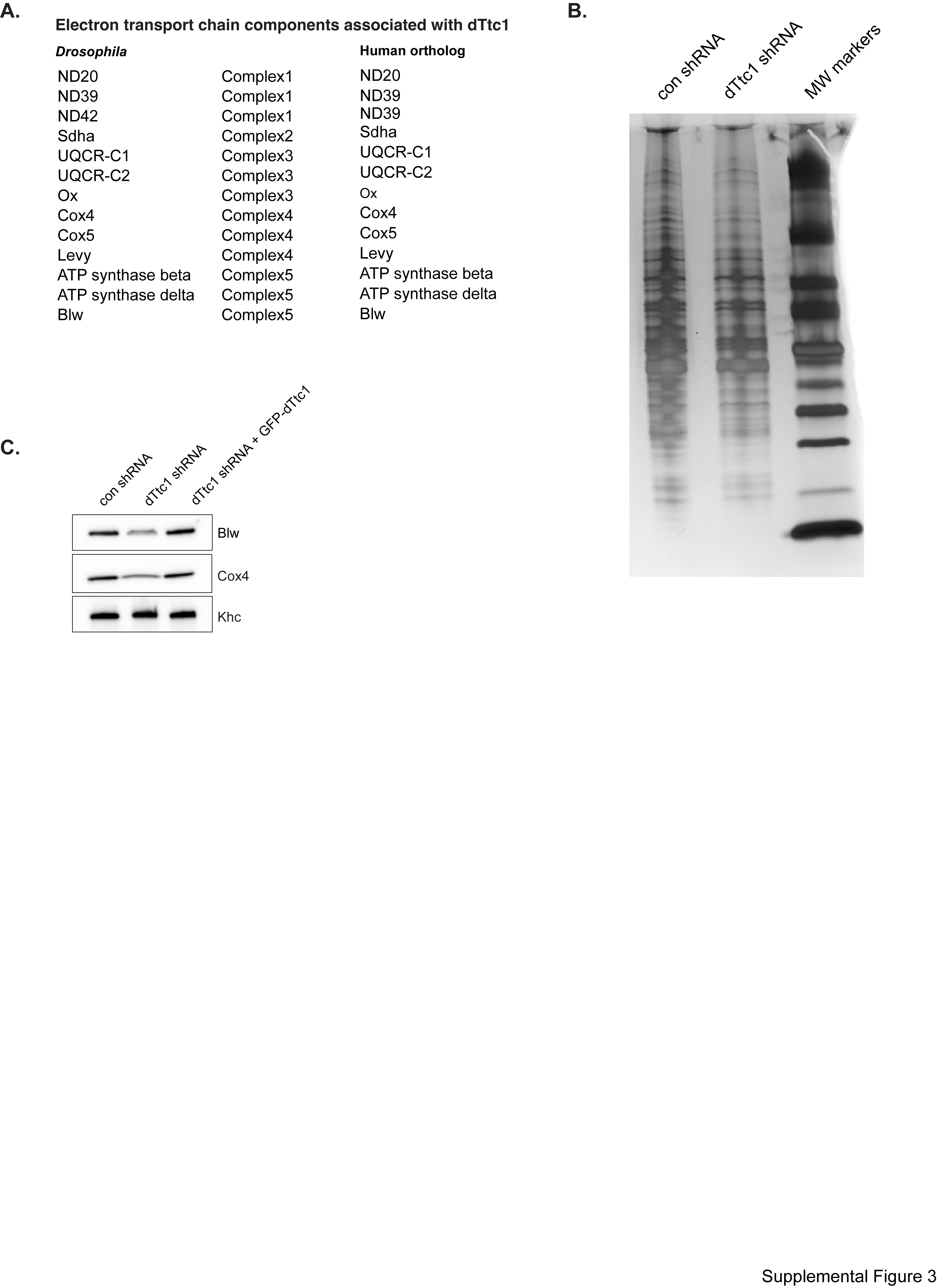

Supplement: Supplementary file 1 [file Image3.TIF]

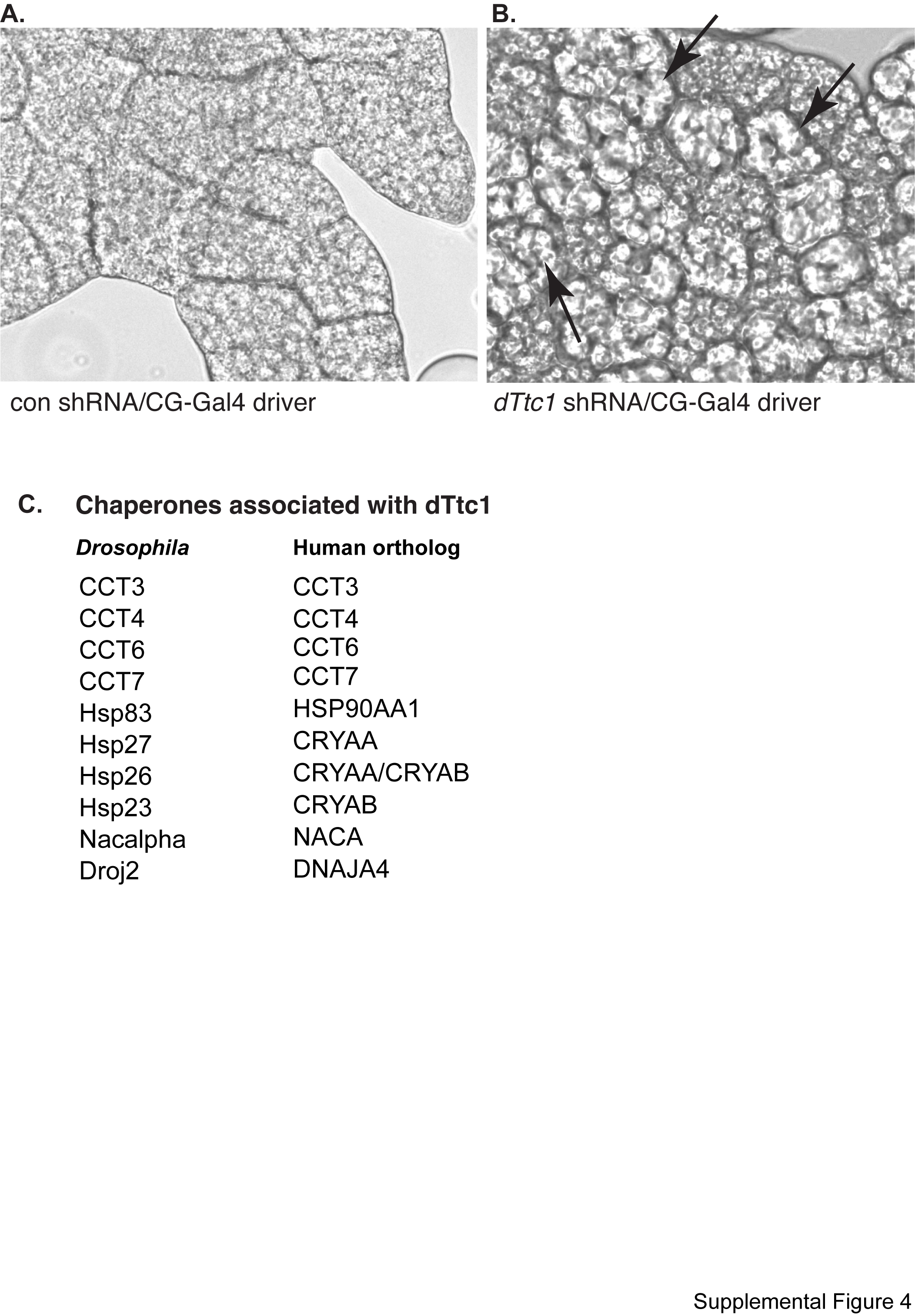

Supplement: Supplementary file 2 [file Image4.TIF]

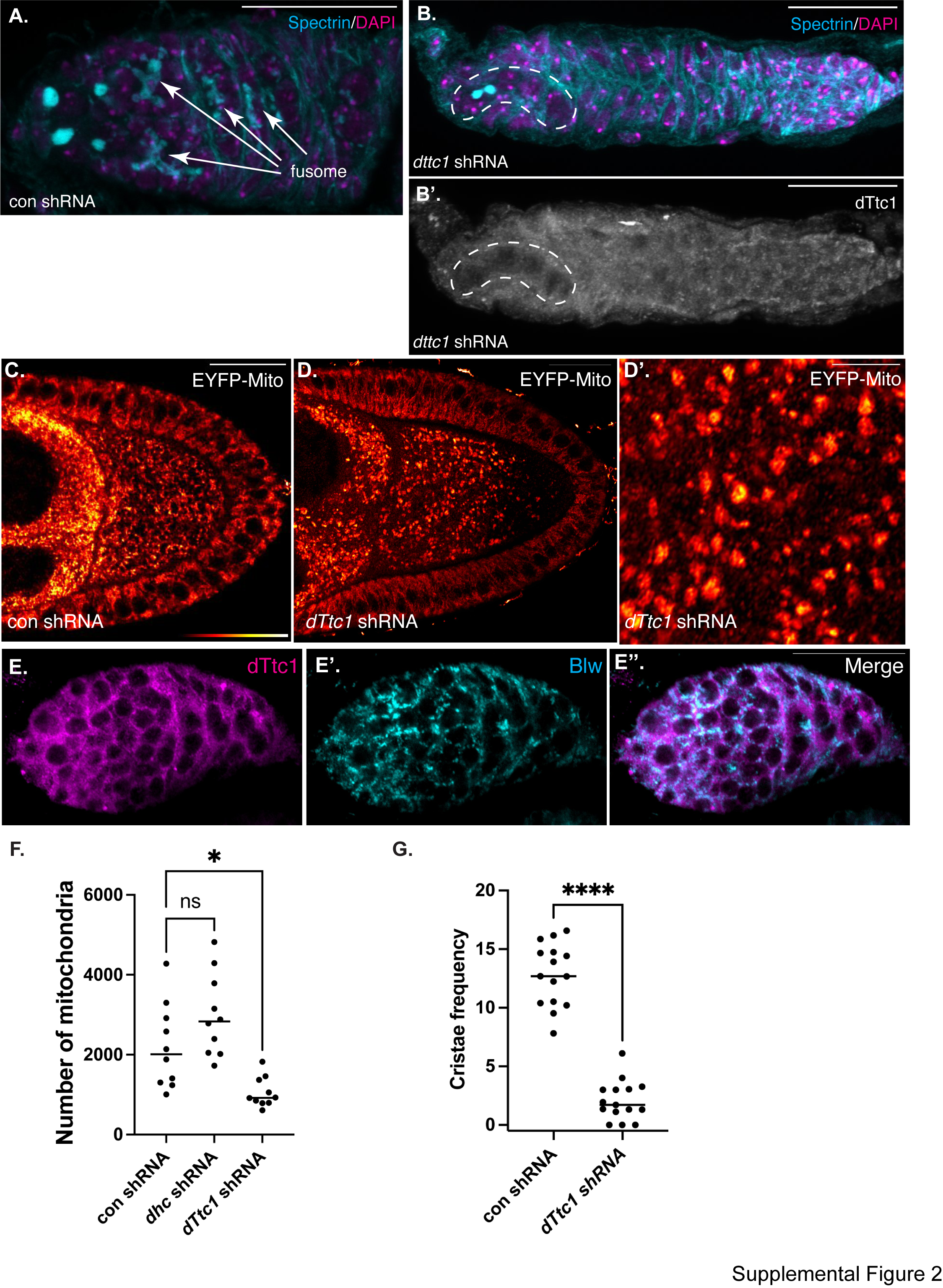

Supplement: Supplementary file 3 [file Image2.TIF]

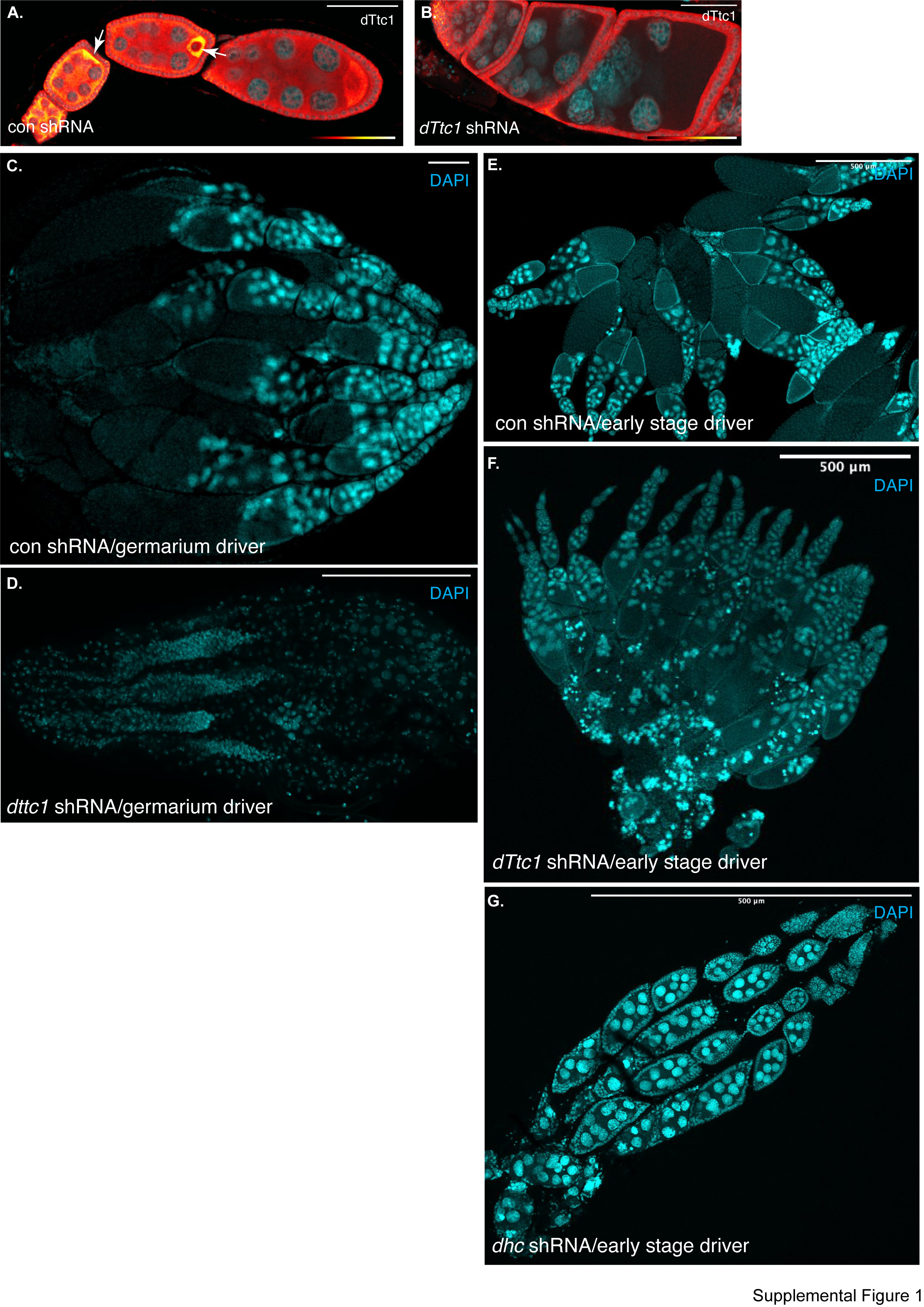

Supplement: Supplementary file 4 [file Image1.TIF]
